# Urban versus rural residency and pancreatic cancer survival: A Danish nationwide population-based cohort study

**DOI:** 10.1371/journal.pone.0202486

**Published:** 2018-08-16

**Authors:** Jakob Kirkegård, Morten Ladekarl, Claus Wilki Fristrup, Carsten Palnæs Hansen, Mogens Sall, Frank Viborg Mortensen

**Affiliations:** 1 Department of Surgery, HPB section, Aarhus University Hospital, Aarhus, Denmark; 2 Department of Oncology, Aarhus University Hospital, Aarhus, Denmark; 3 Odense Pancreas Center (OPAC), Department of Surgery, Odense University Hospital, Odense, Denmark; 4 Department of Surgical Gastroenterology and Transplantation, Rigshospitalet, Copenhagen, Denmark; 5 Department of Surgery, Aalborg University Hospital, Aaborg, Denmark; University of Toronto, CANADA

## Abstract

It is unknown whether urban versus rural residency affects pancreatic cancer survival in a universal tax-financed healthcare system. We conducted a nationwide, population-based cohort study of all patients diagnosed with pancreatic cancer in Denmark from 2004–2015. We used nationwide registries to collect information on characteristics, comorbidity, cancer-directed treatment, and vital status. We followed the patients from pancreatic cancer diagnosis until death, emigration, or 1 October 2017, whichever occurred first. We truncated at five years of follow up. We stratified patients into calendar periods according to year of diagnosis (2004–2007, 2008–2011, and 2012–2015). We used Cox proportional hazards model to compute hazard ratios (HRs) with associated 95% confidence intervals (CIs) of death, comparing patients in urban and rural areas. HRs were adjusted for age, sex, comorbidity, tumor stage, and localization. In a sub-analysis, we also adjusted for cancer-directed treatment. We included 10,594 patients diagnosed with pancreatic cancer. Median age was 71 years (inter-quartile range: 63–78 years), and half were men. The majority (61.7%) lived in an urban area at the time of diagnosis. When adjusting for potential confounders, we observed a better survival rate among pancreatic cancer patients residing in urban areas compared with rural areas (adjusted HR: 0.92; 95% CI: 0.87–0.98). When taking treatment into account, the association was unclear (adjusted HR: 0.96; 95% CI: 0.88–1.04). Pancreatic cancer patients residing in urban areas had a slightly better survival rate compared with patients in rural areas.

## Introduction

Pancreatic cancer accounts for 330,000 deaths globally each year, posing a major healthcare challenge [1, 2]. Pancreatic ductal adenocarcinoma, which is associated with a particularly poor prognosis, constitutes approximately 85% of all pancreatic cancers [3]. In recent years, the prognosis of many different types of cancers has improved considerably. However, despite extensive research, the prognosis of pancreatic cancer still remains dismal and with few advances [4].

The poor prognosis may in part be due to our lack of understanding of this type of cancer, including determinants of its prognosis. Several demographic and clinical factors such as age, tumor histology, and lymph node status as well as tumor size, stage, and location are established prognostic factors [5]. However, non-clinical factors may also affect pancreatic cancer survival. Several studies have examined the association between rural or urban residency and pancreatic cancer survival [6–17]. However, findings have been disparate, and the studies have been conducted in countries with diverse healthcare systems, limiting generalizability. Only two reports—with different results—on this association within a tax-financed healthcare system exist [12, 16]. One of these studies, however, was restricted to patients undergoing resection [12]. The other study had no information on tumor stage or cancer-directed treatment [16]. As such, the association between urban or rural residency and pancreatic cancer survival in a universal tax-financed healthcare system needs clarification.

We therefore conducted a population-based cohort study of patients diagnosed with pancreatic cancer in Denmark, one of the most financially equal countries in the world, measured by the Gini coefficient [18], and a country with universal tax-financed healthcare, to examine the impact of urban versus rural residency on prognosis overall and by different calendar periods.

## Materials and methods

### Setting

We conducted a nationwide, population-based cohort study of all patients diagnosed with pancreatic cancer in Denmark during the period 2004–2015. Individual-level data linkage between Danish healthcare and population-based registries was available using the Civil Personal Registration (CPR) number. The CPR number is a unique identification number assigned to all Danish residents at birth or immigration, allowing virtually complete follow up.

### Study population

We used the Danish Cancer Registry, which was established in 1943 and includes information on all cancers diagnosed in Denmark [19, 20], to identify all patients diagnosed with pancreatic cancer from 1943 through 2015 (n = 30,953). This registry contains information on, among other variables, date of diagnosis, cancer site, histology, and since 2004 tumor-node-metastasis (TNM) classification.(19) Pancreatic cancer patients were identified using *International Classification of Diseases* (ICD) 10^th^ revision code C25.0-C25.9. We excluded all patients diagnosed prior to 2004 (n = 19,584), patients with tumors in situ (n = 17), patients with histologically verified neuroendocrine, cystic, or stromal tumors in addition to lymphomas and sarcomas (n = 385), and patients aged less than 18 years old at the date of pancreatic cancer diagnosis (n = 2). A detailed flowchart and morphology codes are available in S1 and S2 Tables. We used information on the TNM stage to derive the American Joint Committee on Cancer (AJCC) stage. Based on the date of pancreatic cancer diagnosis, we stratified our study population into three calendar periods (2004–2007, 2008–2011, and 2012–2015).

### Information on area of residence

From the Danish Cancer Registry, we also retrieved information on residential municipality at the time of cancer diagnosis. Denmark is divided into five regions and 98 municipalities. We used the official classification from the Danish Ministry of Environment and Food to classify patients into four types of municipalities: 1) remote area municipality, 2) rural municipality, 3) regional municipality, and 4) metropolitan municipality. This is described in more detail elsewhere [21]. In short, this classification is based on 14 indicators and includes, among others, population size and density, proportion of population living in farmlands, distance to major highways, demography, number of workplaces, and socio-economic status. We further grouped patients living in remote areas and rural municipalities into the category “rural areas”, and patients residing in regional and metropolitan municipalities into the category “urban areas”. In general, Danish urban areas have a population density of >100 persons per square-kilometers. The Danish population is approximately 5.5 million inhabitants. Of these, around 2 million lives in a rural area. All areas are comparable with respect to unemployment rates. We excluded patients residing in Greenland and patients with an unknown municipality code (n = 124).

### Information on comorbidity and cancer-directed treatment

We used the Danish National Patient Registry to retrieve a full medical history on each patient in the study population. The Danish National Patient Registry was established in 1977 and contains information on all Danish inpatient hospitalizations [22]. Outpatient and emergency room visits are registered since 1995. Patients are registered in the Danish National Patient Registry with diagnoses according to the ICD 8^th^ revision (ICD-8) from 1977–1993 and ICD 10^th^ revision (ICD-10) hereafter. We used each patient’s medical history registered in the year preceding their pancreatic cancer diagnosis to calculate the Charlson Comorbidity Index (CCI) score [23]. We defined three levels of comorbidity: Low (CCI score 0), moderate (CCI score 1–2), and severe (CCI score >2) comorbidity (ICD codes are available in S3 Table). We excluded non-melanoma skin cancers and pancreatic cancers from the CCI score.

From the Danish National Patient Registry, we also retrieved information on cancer-directed treatment for each patient. This information has been recorded in the registry since 2001. We defined cancer-directed treatment as any pancreatic resection, chemotherapy, or radiation therapy initiated within 120 days after pancreatic cancer diagnosis. Furthermore, we considered all treatments initiated within 30 days prior to pancreatic cancer diagnosis as cancer-directed treatment to allow for late registration of the cancer. We defined seven categories of treatment: 1) best supportive care (no treatment), 2) resection, 3) resection and neoadjuvant therapy, 4) resection and adjuvant therapy, 5) chemotherapy, 6) radiation therapy, and 7) chemoradiation therapy. A full list of ICD codes used to identify the treatments is provided in S4 Table.

### Follow-up

We retrieved information on vital status for each patient from the Civil Registration System, which was established in 1968. The Civil Registration System is an administrative registry containing data on variables like birth date, sex, dates of migration, and vital status for every legal resident in Denmark [24, 25]. We followed each subject from date of pancreatic cancer diagnosis until death, emigration, or October 1, 2017, whichever occurred first. As we examined survival trends over time, we truncated the follow-up periods to five years. Patients who died at the date of pancreatic cancer diagnosis were excluded (n = 247).

### Statistical analyses

We tabulated descriptive characteristics for the study population. Continuous variables are presented as medians with interquartile ranges (IQRs). We computed the median survival in months and 1-, 3-, and 5-year survival for patients residing in rural and urban areas in each calendar period, accounting for censoring. To estimate the impact of geographical residency on overall survival, we fitted a Cox proportional hazards model. We computed crude and adjusted hazard ratios (HRs) with corresponding 95% confidence intervals (CIs) of death, comparing patients in urban areas with patients in rural areas. In the multivariable models, HRs were adjusted for age, sex, CCI score, year of pancreatic cancer diagnosis, tumor location (head, body, tail, and other/multiple regions), and AJCC stage. Patients with missing values of tumor location or AJCC stage were omitted from the Cox models. We conducted two sub-analyses. First, to account for possible variations in which treatment was offered to patients in rural versus urban areas, we started follow-up 120 days after pancreatic cancer diagnosis, and included treatment as a covariate in the Cox models to estimate the direct, rather than the total, effect of areas on residency on pancreatic cancer survival. Accordingly, patients not surviving the first 120 days following pancreatic cancer diagnosis were omitted from this analysis (n = 5,339). Second, we subdivided patients living in urban areas according to the definition from the Ministry of Environment and Food (*i*.*e*. regional or metropolitan area). The proportional hazards assumption was assessed using log(-log) plots, and the model was considered appropriate. All statistical analyses were performed using Stata 15 (StataCorp LP, College Station, Texas, USA).

### Ethical considerations

This study was approved by the Danish Data Protection Agency (*J*.*nr*. *1-16-02-911-17*). According to Danish law, ethical approval is not required for registry-based studies.

### Role of the funding source

This study was supported by a grant from the Danish Cancer Society (Grant no. *R124-A7521*) to Professor Frank Viborg Mortensen. The funding source had no role in the study design, data collection, analysis and interpretation, writing the report, or in the decision to submit the paper for publication.

## Results

### Descriptive characteristics

We included 10,594 patients with pancreatic cancer (Table 1). The majority (61.7%) lived in urban areas at the time of diagnosis. Median age at diagnosis was 71 years (IQR: 63–78 years), and approximately half (50.1%) were men. There was no difference in age, sex, CCI score, calendar period of diagnosis, tumor location, or AJCC stage between patients in urban and rural areas. Overall, 76.3% had a histologically verified diagnosis of pancreatic ductal adenocarcinoma with no major difference between urban (77.1%) and rural (75.0%) areas. Patients residing in urban areas were slightly more likely to undergo surgery and receive chemotherapy compared with patients in rural areas (Table 1).

**Table 1:** Descriptive characteristics of 10,594 patients diagnosed with pancreatic cancer in Denmark, 2004–2015.

	Urban areas		Rural areas	
	N = 6,539 (61.7%)		N = 4,055 (38.3%)	
Age, years, median (IQR)	70.8 (63.4–78.4)		70.9 (53.4–78.4)	
*Age group*				
≤65 years	1,944	29.7%	1,194	29.4%
>65–75 years	2,288	35.0%	1,381	34.1%
>75 years	2,307	35.3%	1,480	36.5%
*Sex*				
Men	3,260	49.9%	2,050	50.6%
Women	3,279	50.1%	2,005	49.4%
*Charlson Comorbidity Index score*				
0	5,198	79.5%	3,236	79.8%
1–2	1,081	16.5%	663	16.4%
>2	260	4.0%	156	3.8%
*Period of diagnosis*				
2004–2007	2,005	30.7%	1,273	31.4%
2008–2011	2,294	35.1%	1,368	33.7%
2012–2015	2,240	34.3%	1,414	34.9%
*Location*				
Head	2,955	45.2%	1,905	47.0%
Body	468	7.2%	319	7.9%
Tail	317	4.8%	212	5.2%
Other/multiple regions	441	6.7%	274	6.8%
Missing	2,358	36.1%	1,345	33.2%
*T-stage*				
T1	314	4.8%	199	4.9%
T2	667	10.2%	505	12.5%
T3	1,273	19.5%	805	19.9%
T4	1,774	27.1%	1,024	25.3%
Tx	2,511	38.4%	1,522	37.5%
*N-stage*				
N0	1,081	16.5%	604	14.9%
N1	2,029	31.0%	1,325	32.7%
Nx	3,429	52.4%	2,126	52.4%
*M-stage*				
M0	2,022	30.9%	1,175	29.0%
M1	3,120	47.7%	1,990	49.1%
Mx	1,397	21.4%	890	21.9%
*AJCC stage*				
Stage I	243	3.7%	147	3.6%
Stage II	699	10.7%	411	10.1%
Stage III	652	10.0%	410	10.1%
Stage IV	3,120	47.7%	1,990	49.1%
Missing	1,825	27.9%	1,097	27.1%
*Histologically verified*				
Yes	5,041	77.1%	3,042	75.0%
No	1,498	22.9%	1,013	25.0%
*Treatment*				
Best supportive care	3,278	50.1%	2,216	54.6%
Resection	296	4.5%	194	4.8%
Resection + neoadjuvant therapy	5	0.1%	10	0.3%
Resection + adjuvant therapy	501	7.7%	217	5.4%
Chemotherapy	2,280	34.9%	1,294	31.9%
Radiation therapy	47	0.7%	34	0.8%
Chemoradiation therapy	132	2.0%	90	2.2%

### Pancreatic cancer survival

#### Entire study period

**Fig 1:**
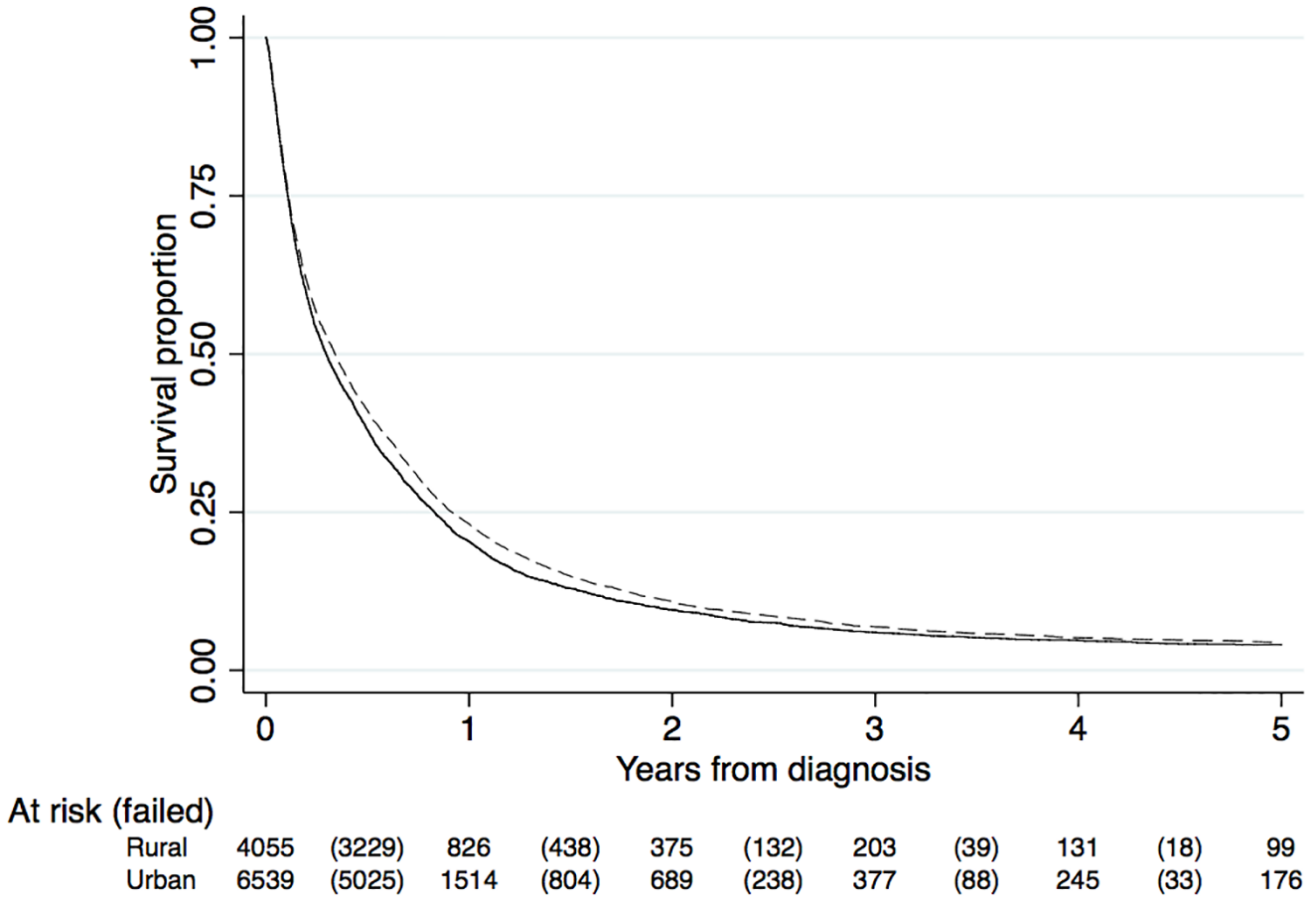
Survival curves for the entire study period. Dashed line: urban residents: full line: rural residents.

For the entire period, median survival time were similar in urban (4.1 months) and rural (3.5 months) areas (Table 2 and Fig 1).

**Table 2:** Survival among 10,594 patients diagnosed with pancreatic cancer in Denmark, 2004–2015.

	Urban areas	Rural areas
	N = 6,539 (61.7%)	N = 4,055 (38.3%)
Median, months (IQR)	4.1 (1.3–11.0)	3.5 (1.2–10.4)
1-year survival (95% CI)	23% (22%-24%)	20% (19%-22%)
3-year survival (95% CI)	7% (6%-8%)	6% (5%-7%)
5-year survival (95% CI)	4% (4%-5%)	4% (3%-5%)
Crude HR (95% CI)	0.94 (0.90–0.97)	*reference*
Adjusted HR^1^ (95% CI)	0.92 (0.87–0.98)	*reference*
Adjusted HR^2^ (95% CI)	0.96 (0.88–1.04)	*reference*

^1^ Adjusted for age, sex, Charlson Comorbidity Index score, year of diagnosis, tumor location, and AJCC stage

^2^ As above, also adjusted for cancer-directed treatment

IQR: interquartile range; CI: confidence interval; HR: hazard ratio

In the Cox model, urban residency was associated with a better overall survival compared with rural residency (HR: 0.94; 95% CI: 0.90–0.97). After adjusting for potential confounders, our overall estimate remained unchanged (adjusted HR: 0.92; 95% CI: 0.87–0.98). After adjustment for cancer-directed treatment, our result suggested that there was no difference between urban and rural areas (adjusted HR: 0.96; 95% CI: 0.88–1.04). Analyses stratified by sex did not alter our findings (results not shown). In our second sub-analysis, metropolitan (adjusted HR: 0.90; 95% CI: 0.85–0.96) but not regional (adjusted HR: 0.96; 95% CI: 0.88–1.04) residency was driving the observed association (S5 Table).

#### Calendar periods

In general, we saw small improvements of median survival time over the study period (Table 3). In the periods 2004–2007 and 2008–2011, we saw no difference in pancreatic cancer survival between urban or rural residents. In the period 2012–2015, we observed that patients in urban areas had a slightly better survival rate compared with patients in rural areas (crude HR: 0.92; 95% CI: 0.85–0.98). This estimate was strengthened after adjustment for potential confounders (adjusted HR: 0.87; 95% CI: 0.79–0.96). After further adjustment for cancer-directed treatment, the association diminished (adjusted HR: 0.92; 95% CI: 0.80–1.05). Our second sub-analysis showed that in the latest calendar period, the observed survival difference was driven by patients living in metropolitan areas, in which the survival advantage was particular pronounced (adjusted HR: 0.85; 95% CI: 0.76–0.95); see S6–S8 Tables.

**Table 3:** Survival among 10,594 patients diagnosed with pancreatic cancer in Denmark, 2004–2015, stratified by calendar period of diagnosis.

	2004–2007		2008–2011		2012–2015	
	Urban areas	Rural areas	Urban areas	Rural areas	Urban areas	Rural areas
	N = 2,005	N = 1,273	N = 2,294	N = 1,368	N = 2,240	N = 1,414
Median, months (IQR)	3.2 (1.2–8.5)	2.9 (1.1–8.8)	4.1 (1.2–12.1)	3.4 (1.0–10.6)	5.1 (1.4–15.6)	4.8 (1.4–13.0)
1-year survival (95% CI)	16% (15%-18%)	15% (13%-17%)	23% (22%-25%)	20% (18%-22%)	29% (27%-31%)	25% (23%-27%)
3-year survival (95% CI)	4% (3%-5%)	4% (3%-5%)	7% (6%-8%)	7% (5%-8%)	10% (8%-11%)	8% (6%-9%)
5-year survival (95% CI)	3% (2%-3%)	3% (2%-4%)	4% (4%-5%)	4% (3%-5%)	6% (5%-7%)	5% (4%-7%)
Crude HR (95% CI)	0.97 (0.90–1.04)	*reference*	0.93 (0.86–0.99)	*reference*	0.92 (0.85–0.98)	*reference*
Adjusted HR^1^ (95% CI)	0.91 (0.82–1.01)	*reference*	0.98 (0.88–1.08)	*reference*	0.87 (0.79–0.96)	*reference*
Adjusted HR^2^ (95% CI)	0.93 (0.80–1.08)	*reference*	1.00 (0.88–1.15)	*reference*	0.92 (0.80–1.05)	*reference*

^1^ Adjusted for age, sex, Charlson Comorbidity Index score, year of diagnosis, tumor location, and AJCC stage

^2^ As above, also adjusted for cancer-directed treatment

IQR: interquartile range; CI: confidence interval; HR: hazard ratio

## Discussion

Findings from this large population-based study suggest that urban—compared with rural—residency is associated with a slightly better survival in patients diagnosed with pancreatic cancer in a universal tax-financed healthcare system. Our association diminished after adjusting for cancer-directed treatment. Our second sub-analysis further suggests that this survival advantage seen in urban patients is driven by patients living in metropolitan areas.

The survival difference observed between urban and rural areas may have several explanations. First, differences in access to, or delivery of, care for pancreatic cancer patients may be different between urban and rural areas as suggested by our sub-analysis. In Denmark, pancreatic cancer surgery is centralized to four centers, whereas oncological treatment is more widely offered. However, there are no differences between survival following pancreatic cancer resection between the four surgical centers in Denmark [26]. Given that Denmark is a small country with limited travel distance between hospitals, free and equal access to healthcare, and reimbursement for some travel expenses to the hospitals we find this finding to be surprising. Differences in health behavior may also contribute to our findings. It is likely that patients living in urban areas are more likely to seek medical attention compared with patients in rural areas, leading to prompter diagnosis of their pancreatic cancer without an actual survival difference (*i*.*e*. lead time bias). However, we did not see any differences in tumor stage or location between urban and rural residents. Also, patients in rural areas may be more exposed to tobacco smoking and alcohol consumption, which may lead to a higher burden of unregistered comorbidity, impairing survival, and possibly more aggressive tumor biology. Furthermore, on population-level, individuals living in urban areas differ from rural areas with respect to age and sex [27], which could affect survival rates. However, the population distribution between urban and rural areas in our study is identical to that of the entire Danish population. Also, in our study age, sex, and comorbidity level were equally distributed between the two groups and thus unlikely to explain findings.

The observed survival difference between urban and rural residents was evident only in the latest calendar period. Although this may be explained by lead time bias due to improved cancer diagnostics, part of the survival improvements over time may also be explained by recent introduction of adjuvant chemotherapy, more efficient palliative chemotherapy, and improvements in supportive care [28, 29].

The impact of geographical residency on pancreatic cancer survival has been investigated in numerous reports. However, these studies have yielded conflicting results. Some studies found that rural residency was associated with a poorer survival [7, 10, 14, 15], whereas other studies suggested that this was the case for patients living in urban areas [8, 9]. In contrast, some studies found no survival difference between urban and rural areas [11, 13, 17]. This discrepancy may be explained by the diverse healthcare settings that these studies were conducted in, such as the United States of America, Australia, Germany, China, and Belgium. As such, results from one healthcare system may not be applicable to other healthcare settings. This is the largest study to date showing that rural residency is associated with an impaired pancreatic cancer survival rate within a universal tax-financed healthcare system. This association has been examined in another tax-financed healthcare system in Canada. Kagedan *et al*. found no difference in survival based on geographical residency in 469 patients undergoing surgical resection for pancreatic cancer [12]. However, our studies are not comparable, as Kagedan *et al*. examined patients undergoing surgical resection, whereas our study included all patients with pancreatic cancer. In agreement with our findings, a cohort study by Raju *et al*. of 9,221 patients with pancreatic cancer found that patients in rural areas had a poorer survival rate than patients in urban areas [16]. Although our estimates are comparable, Raju *et al*. did not include information on tumor stage or cancer-directed treatment, which are important determinants of pancreatic cancer survival [5].

This study contains several strengths. First, our study was conducted within a very homogenous population with free and equal access to tax-financed healthcare. As such, this is not a selected population. Second, data in the Danish Cancer Registry is generally of a very high quality [20, 30]. Third, our population-based tracking system in the Civil Registration System ensured long-term and virtually complete follow up, leading to robust survival estimates. Fourth, our study was of a sufficient size to allow for precise estimates.

Some limitations should be kept in mind when interpreting our results. First, we had missing information on tumor location or AJCC stage for approximately one-third of the study population. As these values are unlikely to be missing at random (*i*.*e*. patients with more advanced disease or rapid deterioration may be less likely to undergo full clinical workup, including staging), this may cause some residual confounding. However, the distribution of missing values was similar among patients residing in urban and rural areas and we therefore consider the impact of these missing data to be low. Second, we did not have information on performance status, which can affect pancreatic cancer survival [31]. It is possible that patients in rural areas have a poorer performance status at the time of diagnosis due to a lower health awareness, which could have affected our estimates. Third, although socio-economic status is partly accounted for in the classification of rural or urban area of residence, residual confounding from socio-economic status may be present in this study, as the mean income in Denmark is generally higher in urban areas compared with rural areas [27]. Fourth, we lacked information on tumor histology in 24% of the patients. However, these patients were equally distributed between the two exposure groups and are unlikely to have confounded our findings, as it is reasonable to assume that the distributions of pancreatic adenocarcinomas among patients with missing information are similar in the two groups.

Our finding of a slightly better prognosis for pancreatic cancer patients living in urban areas compared with patients in rural areas in one of the most equal countries in the world is interesting and warrants further investigation. Our study adds knowledge to the well-known variations in healthcare utilization and outcome in different demographic groups, even in a homogenous population with free and intended equal access to tax-financed healthcare. Clinicians and policymakers should be aware of the inequalities in pancreatic cancer survival based on area of residency.

In conclusion, our findings suggest that patients with pancreatic cancer residing in urban areas have a slightly improved survival compared with patients residing in rural areas at the time of cancer diagnosis, even in a tax-financed healthcare system with universal access.

## Supporting information

S1 Table Flowchart. (DOCX)

S2 Table Morphology codes. (DOCX)

S3 Table ICD codes in the Charlson Comorbidity Index. (DOCX)

S4 Table Treatment codes. (DOCX)

S5 Table Results from sub-analysis (overall survival). (DOCX)

S6 Table Results from sub-analysis (survival in the period 2004–2007). (DOCX)

S7 Table Results from sub-analysis (survival in the period 2008–2011). (DOCX)

S8 Table Results from sub-analysis (survival in the period 2012–2015). (DOCX)
