# Supplementary figures and images for: Urban versus rural residency and pancreatic cancer survival: A Danish nationwide population-based cohort study
Source: PLoS One. 2018 Aug 16;13(8):e0202486. doi: 10.1371/journal.pone.0202486 (PMC6095589; doi:10.1371/journal.pone.0202486)

**S1 Table. Flowchart.**


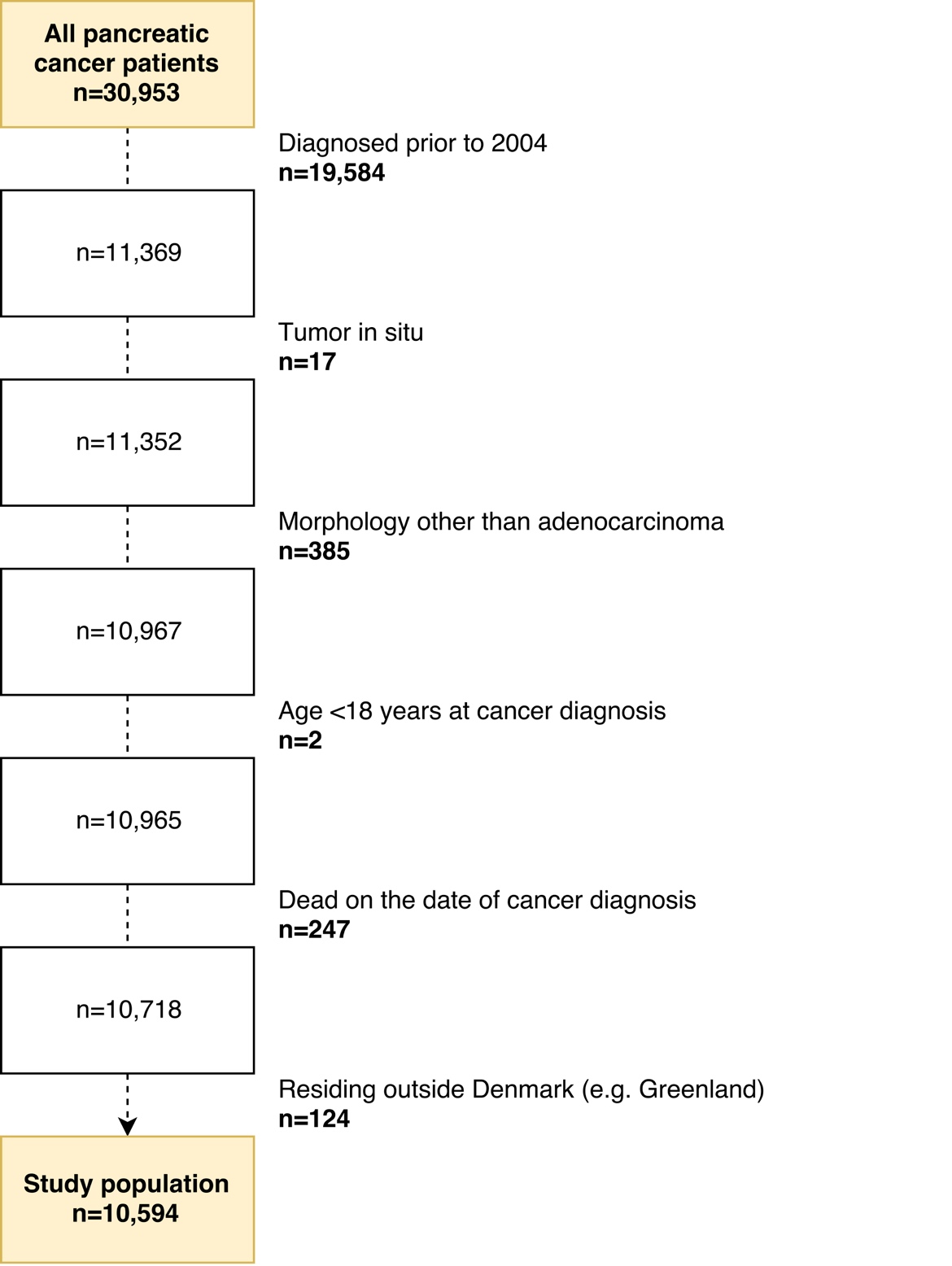

Supplement: S1 Table — (DOCX) [file pone.0202486.s001.docx]
