# Supplementary material for: Urban versus rural residency and pancreatic cancer survival: A Danish nationwide population-based cohort study
Source: PLoS One. 2018 Aug 16;13(8):e0202486. doi: 10.1371/journal.pone.0202486 (PMC6095589; doi:10.1371/journal.pone.0202486)
Supplement: S2 Table — (DOCX) [file pone.0202486.s002.docx]

**S2 Table. Morphology codes.**

| *Pancreatic ductal adenocarcinoma* |
| --- |
| 80003, 80013, 80103, 80123, 80203, 80213, 80223, 80313, 80333, 80353, 81403, 81453, 82303, 82603, 84803, 84903, 85003, 85033, 85603 |
| *Neuroendocrine carcinoma/islet cell carcinoma* |
| 80133, 81503, 81523, 81553, 82403, 82433, 82443, 82463 |
| *Small cell carcinoma* |
| 80413, 80443, 80463 |
| *Squamous cell carcinoma* |
| 80703, 80713, 80723, 80833 |
| *Cholangiocarcinoma* |
| 81603 |
| *Clear cell carcinoma* |
| 83103 |
| *Cystic tumors* |
| 84303, 84403, 84413, 84523, 84533, 84703, 84713 |
| *Acinar cell carcinoma* |
| 85503 |
| *Sarcoma/stroma tumors* |
| 87433, 88003, 89013, 89333, 89363, 91303 |
| *Unknown histology* |
| 99903, 99993 |
