# Supplementary material for: Urban versus rural residency and pancreatic cancer survival: A Danish nationwide population-based cohort study
Source: PLoS One. 2018 Aug 16;13(8):e0202486. doi: 10.1371/journal.pone.0202486 (PMC6095589; doi:10.1371/journal.pone.0202486)
Supplement: S3 Table — (DOCX) [file pone.0202486.s003.docx]

# S3 Table. ICD codes in the Charlson Comorbidity Index.

| **Condition** | | **ICD-8** | | **ICD-10** |
| --- | --- | --- | --- | --- |
| *Score 1* | |  | |  |
|  | Myocardial infarction | 410 | I21-I23 | |
|  | Congestive heart failure | 427.09-427.19, 428.99, 782.49 | I50, I11.0, I13.0, I13.2 | |
|  | Peripheral vascular disease | 440-445 | I70-I74, I77 | |
|  | Cerebrovascular disease | 430-438 | I60-I69, G45, G46 | |
|  | Dementia | 290, 293.09 | F00-F03, F05.1, G30 | |
|  | Chronic pulmonary disease | 490-493, 515-518 | J40-J47, J60-J67, J68.4, J70.1, J70.3, J84.1, J92.0, J96.1, J98.2, J98.3 | |
|  | Connective tissue disease | 712, 716, 734, 446, 135.99 | M05, M06, M08, M09, M30-M36, D86 | |
|  | Ulcer disease | 530.91, 530.98, 531-534 | K22.1, K25-K28 | |
|  | Mild liver disease | 571, 573.01, 573.04 | B18, K70.0-K70.3, K70.9, K71, K73, K74, K76.0 | |
|  | Diabetes types 1 and 2 | 249.00, 249.06, 249.07, 249.09, 250.00, 250.06, 250.07, 250.09 | E10.0, E10.1, E10.9, E11.0, E11.1, E11.9 | |
| *Score 2* | |  |  | |
|  | Hemiplegia | 344 | G81, G82 | |
|  | Moderate to severe renal disease | 403, 404, 580-584, 590.09, 593.19, 753.10-753.19, 792 | I12, I13, N00-N05, N07, N11, N14, N17-N19, Q61 | |
|  | Diabetes with end-organ damage | 249.01-249.05, 249.08, 250.01-250.05, 250.08 | E10.2-E10.8, E11.2-E11.8 | |
|  | Any tumor (except skin and pancreatic cancer) | 140-194 | C00-C75 | |
|  | Leukemia | 204-207 | C91-C95 | |
|  | Lymphoma | 200-203, 275.59 | C81-C85, C88, C90, C96 | |
| *Score 3* | |  |  | |
|  | Moderate to severe liver disease | 070.00, 070.02, 070.04, 070.06, 070.08, 573.00, 456.00-456.09 | B15.0, B16.0, B16.2, B19.0, K70.4, K72, K76.6, I85 | |
| *Score 6* | |  |  | |
|  | Metastatic solid tumor | 195-199 | C76-C80 | |
|  | AIDS | 079.83 | B21-B24 | |
