# Supplementary material for: Urban versus rural residency and pancreatic cancer survival: A Danish nationwide population-based cohort study
Source: PLoS One. 2018 Aug 16;13(8):e0202486. doi: 10.1371/journal.pone.0202486 (PMC6095589; doi:10.1371/journal.pone.0202486)
Supplement: S4 Table — (DOCX) [file pone.0202486.s004.docx]

# S4 Table. Treatment codes.

| **Treatment** | **Code** |
| --- | --- |
| Resection | 48380, 48410, 48420, 48460, 48480, 48500, 48550, 48880, 48890; KJLC10, KJLC20, KJLC30, KJLC40 |
| Chemotherapy | BWHA1, BWHA2 |
| Radiation therapy | BWGC |
