# Supplementary material for: Urban versus rural residency and pancreatic cancer survival: A Danish nationwide population-based cohort study
Source: PLoS One. 2018 Aug 16;13(8):e0202486. doi: 10.1371/journal.pone.0202486 (PMC6095589; doi:10.1371/journal.pone.0202486)
Supplement: S5 Table — (DOCX) [file pone.0202486.s005.docx]

**S5 Table. Results from the sub-analysis (overall survival).**

|  | **Metropolitan**  **N=4,865** | **Regional**  **N=1,674** | **Rural**  **N=4,055** |
| --- | --- | --- | --- |
| Median, months (IQR) | 4.0 (1.3-10.7) | 4.3 (1.3-12.5) | 3.5 (1.2-10.4) |
| 1-year survival (95% CI) | 23% (22%-24%) | 24% (22%-26%) | 20% (19%-22%) |
| 3-year survival (95% CI) | 7% (6%-7%) | 7% (6%-9%) | 6% (5%-7%) |
| 5-year survival (95% CI) | 4% (4%-5%) | 5% (4%-6%) | 4% (3%-5%) |
| Crude HR (95% CI) | 0.95 (0.91-0.99) | 0.90 (0.85-0.95) | *reference* |
| Adjusted HR^1^ (95% CI) | 0.90 (0.85-0.96) | 0.96 (0.89-1.04) | *reference* |
| Adjusted HR^2^ (95% CI) | 0.95 (0.87-1.04) | 0.96 (0.86-1.07) | *reference* |

^1^ Adjusted for age, sex, Charlson Comorbidity Index score, year of diagnosis, tumor location, and AJCC stage

^2^ As above, also adjusted for cancer-directed treatment

IQR: interquartile range; CI: confidence interval; HR: hazard ratio
