# Supplementary material for: Urban versus rural residency and pancreatic cancer survival: A Danish nationwide population-based cohort study
Source: PLoS One. 2018 Aug 16;13(8):e0202486. doi: 10.1371/journal.pone.0202486 (PMC6095589; doi:10.1371/journal.pone.0202486)
Supplement: S6 Table — (DOCX) [file pone.0202486.s006.docx]

**S6 Table. Results from the sub-analysis (survival in the period 2004-2007).**

|  | **Metropolitan**  **N=1,519** | **Regional**  **N=486** | **Rural**  **N=1,273** |
| --- | --- | --- | --- |
| Median, months (IQR) | 3.2 (1.1-8.5) | 3.0 (1.1-10.0) | 2.9 (1.1-8.8) |
| 1-year survival (95% CI) | 15% (14%-18%) | 17% (14%-20%) | 15% (13%-17%) |
| 3-year survival (95% CI) | 4% (3%-5%) | 4% (3%-6%) | 4% (3%-5%) |
| 5-year survival (95% CI) | 2% (2%-3%) | 3% (2%-5%) | 3% (2%-5%) |
| Crude HR (95% CI) | 0.98 (0.91-1.05) | 0.94 (0.85-1.05) | *reference* |
| Adjusted HR^1^ (95% CI) | 0.90 (0.80-1.00) | 0.94 (0.81-1.09) | *reference* |
| Adjusted HR^2^ (95% CI) | 0.91 (0.78-1.06) | 0.95 (0.76-1.19) | *reference* |

^1^ Adjusted for age, sex, Charlson Comorbidity Index score, tumor location, AJCC stage

^2^ As above, also adjusted for cancer-directed treatment

IQR: interquartile range; CI: confidence interval; HR: hazard ratio
