# Supplementary material for: Urban versus rural residency and pancreatic cancer survival: A Danish nationwide population-based cohort study
Source: PLoS One. 2018 Aug 16;13(8):e0202486. doi: 10.1371/journal.pone.0202486 (PMC6095589; doi:10.1371/journal.pone.0202486)
Supplement: S7 Table — (DOCX) [file pone.0202486.s007.docx]

**S7 Table. Results from the sub-analysis (survival in the period 2008-2011).**

|  | **Metropolitan**  **N=1,693** | **Regional**  **N=601** | **Rural**  **N=1,368** |
| --- | --- | --- | --- |
| Median, months (IQR) | 4.1 (1.2-11.9) | 4.1 (1.2-13.4) | 3.4 (1.0-10.6) |
| 1-year survival (95% CI) | 23% (21%-25%) | 25% (22%-29%) | 20% (18%-22%) |
| 3-year survival (95% CI) | 7% (6%-8%) | 8% (6%-11%) | 7% (5%-8%) |
| 5-year survival (95% CI) | 4% (3%-5%) | 5% (3%-7%) | 4% (3%-5%) |
| Crude HR (95% CI) | 0.94 (0.88-1.01) | 0.89 (0.80-0.98) | *reference* |
| Adjusted HR^1^ (95% CI) | 0.97 (0.87-1.07) | 0.99 (0.86-1.13) | *reference* |
| Adjusted HR^2^ (95% CI) | 1.04 (0.90-1.20) | 0.95 (0.78-1.15) | *reference* |

^1^ Adjusted for age, sex, Charlson Comorbidity Index score, year of diagnosis, tumor location, and AJCC stage

^2^ As above, also adjusted for cancer-directed treatment

IQR: interquartile range; CI: confidence interval; HR: hazard ratio
