# Supplementary material for: Urban versus rural residency and pancreatic cancer survival: A Danish nationwide population-based cohort study
Source: PLoS One. 2018 Aug 16;13(8):e0202486. doi: 10.1371/journal.pone.0202486 (PMC6095589; doi:10.1371/journal.pone.0202486)
Supplement: S8 Table — (DOCX) [file pone.0202486.s008.docx]

**S8 Table. Results from the sub-analysis (survival in the period 2012-2015).**

|  | **Metropolitan**  **N=1,653** | **Regional**  **N=587** | **Rural**  **N=1,414** |
| --- | --- | --- | --- |
| Median, months (IQR) | 4.7 (1.3-16.2) | 6.2 (1.4-15.7) | 4.8 (1.4-13.0) |
| 1-year survival (95% CI) | 29% (27%-2531 | 29% (26%-33%) | 25% (23%-27%) |
| 3-year survival (95% CI) | 10% (8%-11%) | 10% (7%-12%) | 8% (6%-9%) |
| 5-year survival (95% CI) | 6% (5%-8%) | 5% (3%-8%) | 5% (4%-7%) |
| Crude HR (95% CI) | 0.93 (0.86-1.00) | 0.89 (0.80-0.98) | *reference* |
| Adjusted HR^1^ (95% CI) | 0.85 (0.76-0.95) | 0.93 (0.81-1.07) | *reference* |
| Adjusted HR^2^ (95% CI) | 0.90 (0.77-1.04) | 0.97 (0.81-1.17) | *reference* |

^1^ Adjusted for age, sex, Charlson Comorbidity Index score, year of diagnosis, tumor location, and AJCC stage

^2^ As above, also adjusted for cancer-directed treatment

IQR: interquartile range; CI: confidence interval; HR: hazard ratio
